# Digital and Remote Health for Older Adults in Rural or Underserved Settings: Systematic Review of Clinical, Behavioral, and Implementation Outcomes

**DOI:** 10.2196/78913

**Published:** 2026-04-21

**Authors:** Víctor Beltrán, Alain Chaple Gil, Constanza Morales-Gómez, Vanessa Campos-Bijit, Rafael Contador, Leonardo Díaz, Jaime Bustos, Eduardo Fernandez

**Affiliations:** 1 Clinical Investigation and Dental Innovation Center (CIDIC), Faculty of Dentistry and Center for Translational Medicine (CEMT-BIOREN), Universidad de La Frontera, Temuco, Chile; 2 Centro de Excelencia de Física e Ingeniería en Salud (CFIS), Universidad de La Frontera, Temuco, Chile; 3 Facultad de Ciencias de la Salud, Universidad Autónoma de Chile, Santiago, Chile; 4 Department of Pathology and Oral Medicine, Faculty of Dentistry, University of Chile, Santiago, Chile; 5 Laboratory of Nanobiomaterials, Research Institute of Dental Sciences, Faculty of Dentistry, University of Chile, Santiago, Chile, 56 229785073, 56 29785073; 6 Millennium Nucleus for Bioproducts, Genomics and Environmental Microbiology (BioGEM), Valparaíso, Chile; 7 Department of Restorative Dentistry, Faculty of Dentistry, University of Chile, Olivos 943, Santiago, 7500505, Chile, +56 2 2978 5050; 8 Department of Stomatology, Faculty of Dentistry, Universidad de Sevilla, Sevilla, Spain; 9 Perioplastic Institute, Santiago, Chile; 10 Department of Prosthodontics, Faculty of Dentistry, University of Chile, Santiago, Chile; 11 Department of Industrial and System Engineering, Universidad de La Frontera, Temuco, Chile; 12 Instituto de Ciencias Biomédicas, Universidad Autónoma de Chile, Santiago, Chile

**Keywords:** older adults, rural health, digital health, telemedicine, remote care, underserved populations, health disparities

## Abstract

**Background:**

Older adults in rural or underserved settings face persistent access barriers. Digital and remote health interventions may mitigate these gaps.

**Objective:**

The aim of this study is to assess technologies, effectiveness, and implementation challenges of digital or remote health interventions for adults ≥60 years old in rural or underserved contexts.

**Methods:**

A systematic search was conducted in 4 electronic databases, such as PubMed, Scopus, Web of Science, and Embase (inception: April 2025), under dual screening by 2 reviewers. The tool Cochrane Risk of Bias 2.0 was used to assess the risk of bias for randomized trials, and Risk Of Bias In Non-randomized Studies–of Interventions (ROBINS-I) was used for nonrandomized trials. For observational studies, we used the National Institutes of Health quality assessment tool, and a narrative synthesis was performed.

**Results:**

Fourteen studies met the inclusion criteria (randomized and nonrandomized designs; median follow-up ~12 mo). Ten of 14 (71%) reported significant improvements in at least 1 clinical end point (eg, hemoglobin A_1c_, blood pressure, and weight), 11 of 14 (79%) improved behavioral or psychosocial outcomes, and 5 of 9 (56%) reduced hospitalizations or acute episodes. Risk-of-bias concerns most frequently related to missing data and selective reporting. Digital literacy and broadband access were the most consistent barriers, while multicomponent, nurse-supported models achieved higher adherence. Equity-related dimensions (place, gender, and socioeconomic status) were variably reported, with limited attention to language, cultural tailoring, or social capital. No study included a formal cost-effectiveness analysis.

**Conclusions:**

Digital and remote health interventions can benefit older adults in rural and underserved settings by improving selected clinical and behavioral outcomes. However, heterogeneity, small sample sizes, and the absence of economic evaluations temper certainty. Importantly, gaps in equity reporting and persistent barriers to digital inclusion highlight the need for future trials to integrate PROGRESS-Plus (place of residence, race/ethnicity, occupation, gender, religion, education, socioeconomic status, social capital, plus other context-specific determinants of health equity) dimensions, embed cost-effectiveness assessments, and design interventions that are both sustainable and accessible to the most underserved populations.

## Introduction

### Background

The aging of the global population poses unprecedented challenges for health systems, particularly in rural and underserved communities where access to care remains limited [1]. By 2050, it is projected that 1 in 6 people worldwide will be over the age of 65 years, with a significant proportion residing in rural regions [2]. These populations often face geographic, economic, and infrastructural barriers that impede access to timely and adequate health services [3]. Older adults in these regions experience a disproportionate burden of chronic diseases, such as diabetes, heart failure, and hypertension, as well as age-related cognitive decline and social isolation [4].

Digital and remote health interventions, such as telemedicine, mobile health (mHealth) applications, and remote patient monitoring, offer promising avenues to overcome these barriers [5]. These tools can facilitate timely communication with health care providers, enable continuous monitoring of vital signs, and support behavioral interventions aimed at chronic disease management [6]. The integration of such technologies into clinical practice has accelerated, particularly in response to the COVID-19 pandemic, which highlighted the need for resilient and accessible health systems [7].

In this context, telehealth platforms can strengthen provider-to-provider communication, facilitate referral pathways, and support coordination of care across rural and underserved settings.

### Health Care Access Inequities in Rural Aging Populations

Access to health care in rural areas is often hindered by long travel distances, reduced mobility, and limited availability of specialists [8]. These issues are compounded for older adults, who may rely on caregivers or public transportation and experience difficulties navigating health care systems [9]. Studies such as the Informatics for Diabetes Education and Telemedicine (IDEATel) trial have demonstrated that telemedicine interventions can improve glycemic control and cardiovascular risk factors in low-income, underserved older adults [10]. Importantly, this study found that individuals from lower socioeconomic strata benefited as much or more than those from higher income groups, suggesting that tailored interventions can reduce health disparities [11].

Similarly, the Telemedical Interventional Monitoring in Heart Failure 2 (TIM-HF2) trial highlighted the effectiveness of remote patient monitoring in managing heart failure. Adherence to remote monitoring protocols was notably higher in rural residents compared to urban dwellers, especially when interventions were accompanied by patient education and regular contact with health care providers [12]. This suggests that rural patients may value the increased contact and support afforded by digital solutions, particularly when traditional in-person care is less accessible [13-15].

### Barriers to Adoption and Usability Concerns

Despite the potential of digital health tools, several challenges persist. Digital literacy remains a significant barrier among older adults, many of whom have limited experience with smartphones, tablets, or web-based applications [16]. Moreover, infrastructural deficits—such as unreliable broadband access—disproportionately affect rural areas and can hinder the consistent use of telehealth platforms [17].

Usability and accessibility of devices also play a critical role in determining intervention success. The adoption of user-friendly interfaces, simplified monitoring equipment, and culturally tailored content can enhance engagement among older users. DiNapoli et al [18] emphasize the importance of addressing age-related cognitive and sensory impairments when designing digital interventions, noting that failure to do so can limit the reach and effectiveness of these programs.

### Potential for Social and Behavioral Benefits

Beyond clinical outcomes, digital health interventions may also address broader social determinants of health. Perri et al [19] reported that remote interventions helped reduce social isolation and increased self-efficacy in older adults, fostering a sense of autonomy and connectedness. These psychological and behavioral dimensions are particularly important for rural seniors, who often report higher levels of loneliness and depression compared to their urban counterparts [20].

### Rationale for a Systematic Review

Given the rapid expansion of digital health technologies and the pressing needs of aging rural populations, there is an urgent need to synthesize the existing literature to identify effective strategies, common pitfalls, and research gaps. Although some studies have reported promising results, the evidence remains fragmented, and many interventions lack rigorous evaluation. Understanding which approaches work best—and under what conditions—can inform policy decisions and guide the development of inclusive, scalable solutions.

### Objective

This review aimed to synthesize evidence on digital and remote health interventions for adults aged ≥60 years in rural or otherwise underserved settings, describing (1) technologies and care models, (2) clinical, behavioral, and service-use outcomes, and (3) implementation barriers/facilitators through an equity lens. The primary analytic perspective was patient-centered, focusing on clinical, behavioral, and usability outcomes among older adults; provider-facing facilitators and system-level factors were considered as complementary.

## Methods

### Study Design

This systematic review adhered to the PRISMA 2020 [21] (Preferred Reporting Items for Systematic Reviews and Meta-Analyses; CRD420251066174) guidelines and was framed using the PROPS [22] (Population, Remote Health Interventions, Outcomes, Procedures, and Study Design) approach. The PROPS framework provided a structured basis for defining the scope of the review as follows:

- Population: adults aged 60 years or older residing in rural or underserved communities
- Intervention: digital or remote health interventions, including telemedicine, mHealth, wearable devices, and remote monitoring tools
- Outcomes: clinical health parameters (eg, blood pressure and glucose levels), behavioral and psychosocial measures, implementation outcomes (feasibility, adherence, and satisfaction), and digital literacy-related outcomes
- Study design: randomized controlled trials, quasi-experimental studies, prospective and retrospective cohort studies, cross-sectional studies, and mixed methods designs

### Eligibility Criteria

We included peer-reviewed papers published in all idioms until April 2025 that reported on digital or remote health interventions targeting adults aged ≥60 years living in rural or underserved settings. Eligible study designs included randomized controlled trials, quasi-experimental studies, cohort studies, cross-sectional studies, and mixed methods evaluations. Interventions had to utilize telemedicine, mHealth, remote monitoring, or other digital health modalities. Studies exclusively focused on urban populations, institutionalized participants, or individuals under the age of 60 years were excluded.

### Search Strategy

A comprehensive search was conducted in 4 electronic databases: PubMed, Scopus, Web of Science, and Embase. The search strategy combined Medical Subject Headings and free-text terms related to “older adults,” “digital health,” “telemedicine,” “remote monitoring,” “mobile health,” “rural populations,” and “underserved populations.” Search strings were customized for each database. The last search was conducted in April 2025. Reference lists of included papers and relevant reviews were searched manually for additional eligible studies (Multimedia Appendix 1).

### Study Selection

Two independent reviewers (EF and ACG) screened the titles and abstracts using the Rayyan QCRI (Qatar Computing Research Institute) [23] platform. Full texts were retrieved for papers that deemed potentially eligible. Disagreements were resolved through discussion or consultation with a third reviewer (LD). The selection process followed the PRISMA 2020 guidelines [20] (Checklist 1), and a flowchart depicting the number of studies screened, included, and excluded was prepared.

### Data Extraction

A standardized extraction form was developed and piloted. Extracted data included author(s), year of publication, country, study design, sample size, mean participant age, intervention characteristics, technology used, health domain targeted, key outcomes, usability measures, digital literacy considerations, implementation strategies, and barriers and facilitators. Intervention types and target health outcomes were categorized following the framework derived from the included studies. One reviewer (ACG) conducted data extraction, and a second reviewer (EF) independently verified the entries.

### Risk-of-Bias Assessment

Risk of bias was assessed using the Cochrane RoB 2.0 [24] tool for randomized trials and the ROBINS-I [25] tool for nonrandomized studies. For observational studies, we used the NIH quality assessment tool. Two reviewers conducted the assessments independently and discussed discrepancies to reach consensus. Risk-of-bias results were tabulated, and sensitivity analyses were performed when feasible.

### Data Synthesis

Given the heterogeneity of intervention types, outcome measures, and study designs, a narrative synthesis was performed. Studies were grouped according to health conditions targeted (eg, cardiovascular and mental health), types of digital intervention (eg, teleconsultation, mobile app, and remote monitoring), and geographic regions. Themes from qualitative studies were synthesized using thematic analysis and included in cross-cutting narratives. Subgroup comparisons (eg, by rural vs semirural, by intervention duration, or technology type) were conducted where sufficient data were available.

### Synthesis Under the PROPS Framework

Aligned with the PROPS framework, the population (P) included older adults in rural or underserved communities. Remote health interventions (R) varied in format but consistently included a digital or telehealth component. Outcomes (O) encompassed clinical parameters, behavioral change, patient-reported satisfaction, and health care utilization. Procedures (P) typically involved remote coaching, telemonitoring, or integrated care coordination. Study design (S) was predominantly randomized or quasi-experimental, ensuring a moderate to high level of evidence.

### Ethical Considerations

As this study was based entirely on published literature, no ethical approval was required.

## Results

### Characteristics of Included Studies

**Figure 1.**
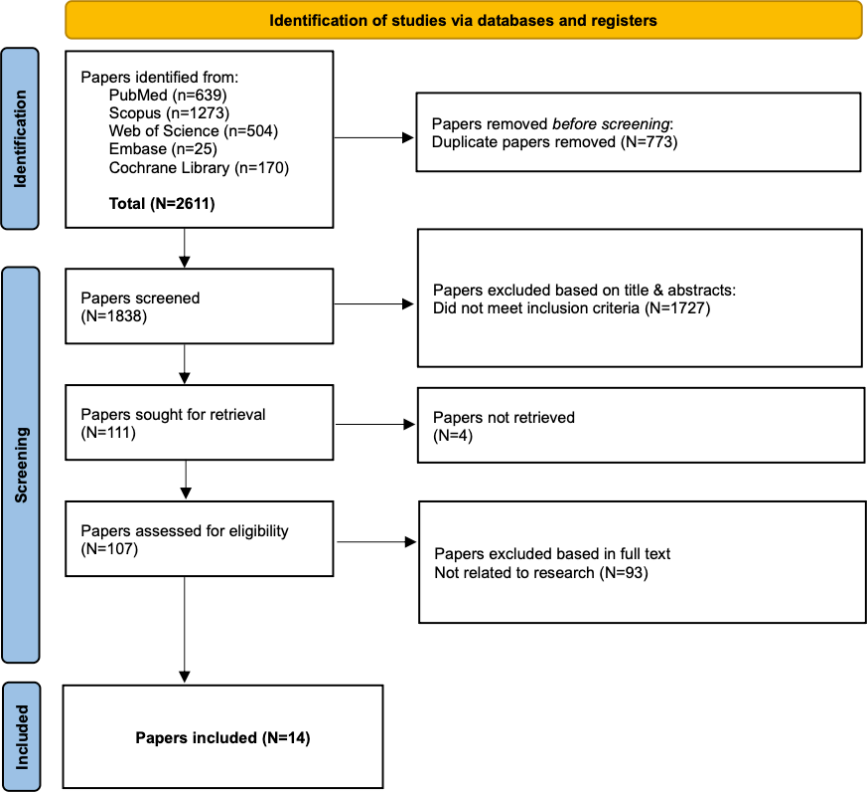
PRISMA 2020 (Preferred Reporting Items for Systematic Reviews and Meta-Analyses 2020) flowchart of study selection process**.** This flow diagram illustrates the identification, screening, eligibility, and inclusion stages of the systematic review. A total of 14 studies met the eligibility criteria and were included in the final synthesis after full-text assessment.

A total of 14 studies met the inclusion criteria and were included in this systematic review (Figure 1). These studies were conducted across diverse settings, including the United States, Canada, Germany, and Australia. The target populations comprised older adults residing in rural or underserved areas and patients with chronic conditions, such as diabetes mellitus, cardiovascular disease, obesity, heart failure, or postsurgical recovery (Table 1). The majority of the interventions combined digital technologies—such as telemonitoring, phone-based coaching, and web-based rehabilitation—with behavioral, educational, or case management support. Table 1 summarizes the characteristics of included studies, Table 2 details intervention types and digital tools employed, and Table 3 presents the reported outcomes and implementation challenges.

**Table 1.** Characteristics of included studies.

Study	Country	Population	Telehealth intervention	Comparator	Main outcomes	Follow-up duration
Barnason et al [16] (2019)	United States	Cardiac patients (post-PCI^a^ or CABS^b^), overweight/obese, rural	12-week phone coaching+nurse support	Usual care	Weight loss and self-efficacy	4 and 6 months
DiNapoli et al [18] (2017)	United States	Older adults, low QoL^c^, mild cognitive impairment, rural	16‐20 sessions of phone-delivered CBT^d^	Minimal support control (weekly calls)	Anxiety, depression, and QoL	6 months
Eberly et al [7] (2025)	United States	Heart failure, underserved	Phone-based GDMT^e^ titration+blood pressure monitoring	Usual care	GDMT uptake	30 days
Krum et al [26] (2013)	Australia	Heart failure, rural	Phone support+nurse follow-up	Usual care	Hospitalizations and mortality	12 months
Lear et al [3] (2015)	Canada	Cardiac patients, rural and small urban	Web-based rehabilitation platform	Usual care	Exercise capacity and lipid profile	16 months
Perri et al [19] (2019)	United States	Obese adults (528), rural	Telecoaching+tracking	In-person	Weight loss and retention	4, 6, and 12 months
Perri et al [17] (2020)	United States	Obese adults (445), rural	Phone counseling (group/individual)+education	Usual care	Weight loss and maintenance	4, 10, 16, and 22 months
Prescher et al [12] (2023)	Germany	Elderly with HF^f^ (1538), rural/urban	Remote monitoring+care coordination	Usual care	Function, QoL, BP^g^, ECG^h^	12 months
Russell et al [27] (2011)	United States	Post-knee arthroplasty patients (65), rural	Telemonitoring	Usual care	Function and OA^i^ Index	6 weeks
Shea et al [4](2009)	United States	Diabetes (1665), underserved	Telemonitoring+nurse case management	Usual care	HbA_1c_^j^, LDL^k^, BP, and satisfaction	5 years
Shea et al [10,28] (2013)	United States	Diabetes, underserved	Telemonitoring+ nurse care management	Usual care	HbA_1c_, SBP^l^, and LDL	12 months
Smith et al [29] (2000)	United States	Rural older adults	Telehealth counseling	Not reported	QoL and satisfaction	5 months
West et al [30] (2010)	United States	Diabetes, rural older adults	Telehealth counseling	Usual care	Behavior change	2‐6 years
Wilson et al [20] (2016)	United States	Smokers, rural	Telemedicine advice	Usual care	Abstinence and self-efficacy	6 months

a PCI: percutaneous coronary intervention.

b CABS: coronary artery bypass grafting.

c QoL: quality of life.

d CBT: cognitive behavioral therapy.

e GDMT: guideline-directed medical therapy.

f HF: heart failure.

g BP: blood pressure.

h ECG: electrocardiogram.

i OA: osteoarthritis.

j HbA_1c_: glycated hemoglobin.

k LDL: low-density lipoprotein.

l SBP: systolic blood pressure.

**Table 2.** Effectiveness outcomes by studies.

Study	Clinical outcome	Behavioral outcome	Patient satisfaction	Health care utilization
Barnason et al [16] (2019)	Sustained weight loss	Improved activity and self-efficacy	Moderate satisfaction	Not reported
DiNapoli et al [18] (2017)	No significant clinical change	Improved mood and activation	High satisfaction	Not reported
Eberly et al (2025)	Increased GDMT^a^ use	Improved adherence	High protocol adherence	Fewer HF^b^ hospitalizations
Krum et al [26] (2013)	Reduced hospitalizations	Not reported	Not reported	Reduced hospitalizations
Lear et al [3] (2015)	Better lipid profile	Not reported	Usability positive	Not reported
Perri et al [19] (2019)	Weight loss	High goal attainment	High satisfaction	Reduced episodes
Perri et al [17] (2020)	Weight loss	Behavior change	High satisfaction	Reduced episodes
Prescher et al [12] (2023)	Cardiac improvements	Improved motivation	High adherence	Lower hospitalization
Russell et al [27] (2011)	Improved OA^c^ outcomes	Self-care adherence	High satisfaction	Reduced episodes
Shea et al [4] (2009)	HbA_1c_^d^, BP^e^ reduction	Empowerment increased	Positive care perception	Lower rates
Shea et al [10,28] (2013)	HbA_1c_, SBP^f^ reduced	Health management	Not reported	Not reported
Smith et al [29] (2000)	No clinical change	Health awareness	Positive experience	Not reported
West et al [30] (2010)	Improved diabetes control	Goal execution improved	High satisfaction	Not reported
Wilson et al [20] (2016)	Verified smoking cessation	Health awareness	High acceptability	Not reported

a GDMT: guideline-directed medical therapy.

b HF: heart failure.

c OA: osteoarthritis.

d HbA_1c_: glycated hemoglobin.

e BP: blood pressure.

f SBP: systolic blood pressure.

**Table 3.** Clinical recommendations.

Clinical domain	Recommended modality	Target population	Recommendation
Chronic conditions	Multicomponent (coaching+monitoring)	Rural older adults	Integrate digital tools with behavioral support
Diabetes	Telemonitoring+nurse coaching	Rural older adults	Combine remote feedback and coaching
Cardiovascular rehabilitation	Web-based rehabilitation+support	Postcardiac discharge, rural	Use online rehabilitation when in-person is not feasible
Weight management	Behavioral telecoaching	Obese rural adults	Support goals via telecoaching
Mental health	Phone-based behavioral therapy	Rural older adults	Implement CBT^a^ remotely

a CBT: cognitive behavioral therapy.

### Clinical Outcomes (Patient Perspective)

Clinical improvements were reported in most of the included studies. Glycemic control (hemoglobin A_1c_ [HbA_1c_]), blood pressure, lipid profiles, and weight reduction were the most frequently evaluated parameters. For example, Shea et al [10,28] observed significant reductions in HbA1c and systolic blood pressure following nurse-led telemonitoring interventions in low-income and underserved diabetic populations. Similarly, Wilson et al [20] reported biochemical validation of smoking abstinence in rural participants receiving telemedicine counseling, whereas Perri et al [17,19] demonstrated sustained weight loss in older obese adults receiving telecoaching and tracking interventions for up to 22 months.

In the cardiovascular domain, Lear et al [3] found that a web-based rehabilitation platform improved lipid profiles and exercise capacity, whereas Krum et al [26], Barnason et al [16], and Prescher et al [12] reported fewer heart failure–related hospitalizations and improvements in weight and blood pressure through structured remote follow-up (Table 2). Notably, Eberly et al [7] demonstrated a significant increase in the uptake of guideline-directed medical therapy for heart failure, supported by remote blood pressure monitoring and medication titration.

### Behavioral and Psychosocial Outcomes

Fifteen studies evaluated behavioral and psychosocial variables, including self-efficacy, adherence, physical activity, and mood. DiNapoli et al [18] used cognitive behavioral therapy (CBT) delivered through telephone and observed improvements in mood, patient activation, and decreased depressive symptoms. Barnason et al [16] and Perri et al [17] reported increases in health-related behaviors, such as dietary control, physical activity, and treatment adherence, alongside weight loss outcomes.

Several studies emphasized the role of motivation, activation, and goal setting. For instance, West et al [30] and Smith et al [29] found that behavioral activation and health awareness were significantly improved among older rural adults participating in structured telehealth counseling sessions. Notably, these effects were more pronounced when interventions included personalized coaching and caregiver engagement.

### Usability and Satisfaction (Patient-Reported)

Patient satisfaction and usability were assessed in 13 studies. Most interventions received favorable feedback from participants, who cited increased accessibility, reduced need for transportation, and personalized interactions as key benefits. Interventions led by nurses or care coordinators (eg, Shea et al [4], Prescher et al [12], and Smith et al [29]) had especially high ratings. Perri et al [19] reported high retention and satisfaction in group-based telecoaching models, emphasizing the value of social support and continuous feedback.

Although some participants noted challenges, such as intervention length or digital interface complexity, overall engagement and willingness to continue using digital health solutions remained strong across studies (Table 2).

### Health Care Utilization and Efficiency

Nine studies reported health care utilization outcomes, including hospital readmissions, acute episode rates, and emergency department visits. Telehealth interventions consistently reduced hospitalization rates in older adults with heart failure or chronic disease, as evidenced in studies by Krum et al [26], Barnason et al [16], and Prescher et al [12]. Shea et al [4] and Perri et al [17] observed reduced acute episodes and improved care coordination through remote monitoring.

While Eberly et al [7] documented fewer cardiac hospitalizations through proactive titration of medications, the majority of studies lacked formal cost-effectiveness analyses. As shown in Table 4, future evaluations must address the economic implications of scaling telehealth models in rural and resource-constrained contexts.

**Table 4.** Limitations and research recommendations.

Limitation	Future research action	Justification
Short follow-up	Long-term RCTs^a^ (≥12 mo)	Assess sustainability
Small samples	Larger trials with stratification	Avoid type II errors
Lack of cost analysis	Include economic evaluations	Guide policy and budgeting
Limited generalizability	Cultural tailoring across regions	Address population heterogeneity
Inconsistent outcomes	Standardized outcome sets	Enable synthesis
Digital barriers	User-centered design for low literacy	Improve accessibility

a RCTs: randomized controlled trials.

### Risk of Bias and Study Quality

**Figure 2.**
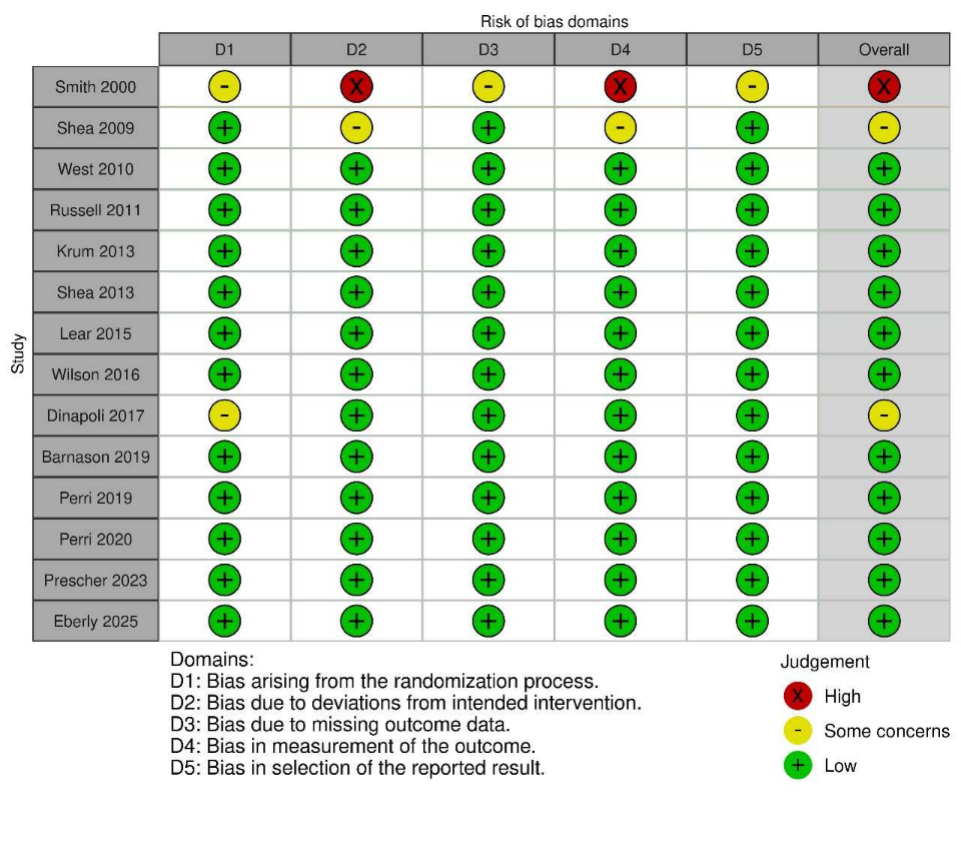
Risk-of-bias assessment for the 14 included studies. Graphical summary of bias domains assessed using the Cochrane Risk of Bias 2.0 tool. Domains include D1 (randomization process), D2 (deviations from intended intervention), D3 (missing outcome data), D4 (measurement of the outcome), and D5 (selection of reported result). Green (+) indicates low risk, yellow (−) indicates some concerns, and red (×) indicates high risk. Overall risk of bias was low in 12 studies, with two studies (Smith et al [29] and DiNapoli et al [18]) rated as high or having some concerns [3,4,7,10,12,16-20,26,27,29,30].

Figure 2 presents the risk of bias assessment. Using the RoB 2.0 tool, most studies were classified as low risk in randomization, outcome measurement, and reporting domains. However, Smith et al [29] was flagged as high risk due to deviation from protocols and unclear allocation procedures, and DiNapoli et al [18] presented some concerns in the randomization process. Despite these limitations, overall study quality was deemed adequate, with the majority of trials employing rigorous designs and clear outcome reporting.

### Implementation Facilitators (Provider Perspective)

Key implementation barriers included limited digital literacy, lack of broadband connectivity, and insufficient cultural adaptation of interventions. Several studies highlighted the importance of preintervention training, simplified user interfaces, and language-appropriate materials. For example, Russell et al [27] and DiNapoli et al [18] integrated patient education modules and caregiver support, which improved both adherence and satisfaction.

Multicomponent interventions—combining synchronous coaching (eg, telephone and video) and asynchronous tracking (eg, mobile apps and remote monitoring)—were associated with greater behavioral change and health gains. Interventions with durations of 6 months or longer demonstrated more durable outcomes. Conversely, short follow-up periods (eg, 30 days in Eberly et al [7]) limited the understanding of long-term sustainability.

### Heterogeneity and Feasibility of Meta-Analysis

The feasibility of conducting a meta-analysis was limited by marked heterogeneity across the included studies in terms of populations, intervention modalities, outcome measures, and follow-up durations. Clinical end points ranged from HbA_1c_ and blood pressure to functional capacity and psychological scales, with inconsistent definitions and reporting formats that precluded statistical pooling. Moreover, small sample sizes and diverse study designs further reduced comparability. For these reasons, a narrative synthesis was judged to be the most appropriate approach, allowing results to be interpreted within outcome domains while acknowledging variability in effect sizes and methodological rigor.

### Synthesis Approach (PROPS)

Findings were organized according to the PROPS framework, grouping evidence by population characteristics, remote health intervention types, outcomes, and setting or contextual factors. This approach allowed us to compare heterogeneous studies in a structured manner and highlight converging patterns. For example, in populations with cardiometabolic conditions, telemonitoring combined with nurse support consistently improved HbA_1c_ and blood pressure outcomes, whereas in mental health or behavioral domains, telephone-based CBT and lifestyle coaching showed greater impact on psychological well-being and adherence. Variability across settings, particularly in broadband access and digital literacy, was emphasized as a determinant of intervention success. Narrative weighting of evidence was guided by risk-of-bias judgments, giving higher emphasis to low-risk trials and cautious interpretation of studies with missing data or selective reporting

### Equity (PROGRESS-Plus)

Equity-related variables, such as place, gender, and socioeconomic status, were variably reported across studies, while domains, such as religion and social capital, were largely absent. Detailed reporting of each dimension and study is provided in Table S4 in Multimedia Appendix 1.

### Summary

Tables 1-4 synthesize the evidence on participants’ characteristics, effectiveness, implementation strategies, and limitations. The findings support the potential of digital health interventions to improve health outcomes, reduce hospitalization, and enhance engagement among older adults in rural or underserved settings. However, sustainability, equity, and generalizability remain key areas for further research.

Comprehensive supplementary data provide additional granularity to the synthesized findings. Table S1 in Multimedia Appendix 1 details the complete database search strategy used across PubMed, Scopus, Web of Science, Embase, and Cochrane, confirming methodological transparency and reproducibility. Table S2 in Multimedia Appendix 1 summarizes the direction, magnitude, and certainty of effects for each included study by outcome domain, strengthening the interpretation of clinical and behavioral improvements reported in the main text. Table S3 in Multimedia Appendix 1 presents the frequency distribution of risk-of-bias domains across randomized, nonrandomized, and observational designs, supporting the overall quality assessment discussed earlier. Equity dimensions were systematically mapped in Table S4 in Multimedia Appendix 1, evidencing variable reporting across PROGRESS-Plus (place of residence, race/ethnicity, occupation, gender, religion, education, socioeconomic status, social capital, plus other context-specific determinants of health equity) categories. Table S5 in Multimedia Appendix 1 provides adherence and attrition data, illustrating high engagement rates (>75%) despite common technical barriers, and Table S6 in Multimedia Appendix 1 classifies intervention models by delivery type, clinical applicability, and observed outcomes, emphasizing the comparative advantages of multicomponent, hybrid telehealth formats. Collectively, these supplementary materials reinforce the robustness, contextual diversity, and implementation relevance of the reviewed evidence.

## Discussion

### Main Findings

This systematic review of 14 studies shows that remote and digital health interventions can significantly improve clinical, behavioral, and satisfaction-related outcomes in older adults living in rural or underserved settings. Telehealth modalities, such as telephone counseling, nurse-led monitoring, and web-based platforms, consistently enhanced chronic disease management, promoted healthier behaviors, and increased accessibility to care. Notable clinical benefits included reductions in HbA_1c_, blood pressure, and low-density lipoprotein cholesterol in diabetic patients, improved uptake of guideline-directed medical therapy and fewer short-term hospitalizations in heart failure, and better weight loss and retention outcomes in obesity. Behavioral interventions also proved effective, with structured coaching and CBT programs improving patient activation, physical activity, and psychological well-being, highlighting the potential of remote care to address barriers faced by these populations.

### Comparison With Prior Work

Our findings align with prior evidence indicating the efficacy of remote interventions in chronic disease management. For instance, Shea et al’s [4] work on the IDEATel project has long emphasized the role of telehealth in improving glycemic and blood pressure control in underserved populations. Similarly, Lear et al [3] confirmed the value of web-based platforms in cardiac rehabilitation, echoing the current study’s results. However, while prior meta-analyses (eg, Marcolino et al [31]) have generally combined urban and rural populations, this review narrows the focus to older adults in rural or resource-limited settings, highlighting unique challenges and opportunities in this demographic.

Several of the studies in this review support the role of multicomponent telehealth strategies that combine behavioral coaching, self-monitoring tools, and clinical oversight. For example, Prescher et al [12] demonstrated improvements in cardiac functions and reduced hospitalizations when remote monitoring was integrated with coordinated care. This convergence of digital tools and human support appears especially important in rural contexts, where health care infrastructure and workforce shortages often limit access to traditional services [14,32].

### Interpretation by Outcome Domains

When interpreting the findings by outcome domains, the evidence suggests that digital and remote health interventions can generate benefits across clinical, behavioral, and health care utilization outcomes, though with varying levels of certainty. Clinical outcomes, such as HbA1c reduction, blood pressure control, and weight loss, were reported in a subset of studies, indicating that structured remote monitoring and nurse-supported programs are particularly effective for cardiometabolic management. Behavioral and psychosocial domains showed improvements in patient activation, medication adherence, and psychological well-being, highlighting the value of tailored telecoaching and CBT-based interventions. In contrast, health service utilization outcomes, such as reduced hospitalizations or emergency visits, were less consistently observed, likely reflecting short follow-up periods and small sample sizes. Taken together, these results indicate that while the strongest evidence lies in clinical and behavioral benefits, sustained improvements in health care utilization may require longer interventions and broader system-level integration.

### Equity Appraisal (PROGRESS-Plus)

Applying the PROGRESS-Plus framework revealed important gaps in how equity dimensions were reported and addressed across the included studies. Most trials provided some data on place of residence, education, or socioeconomic status, but few explicitly measured digital literacy, broadband availability, or cultural and linguistic adaptation of interventions. Gender and age were routinely reported, yet aspects such as occupation, religion, or social capital were rarely considered. Notably, none of the studies systematically evaluated how these factors moderated intervention outcomes, which limits the ability to determine whether digital tools reduced or inadvertently widened disparities. This underreporting underscores the need for future research to integrate equity indicators prospectively into study design and to tailor interventions to the structural barriers and lived experiences of rural and underserved older populations.

### Limitations of Included Studies

Despite promising outcomes, limitations in study design, follow-up duration, and reporting hinder the generalizability of findings. As summarized in Table 4, many studies had short follow-up durations (eg, 30 d to 6 mo), which limit our understanding of long-term intervention sustainability. Only a few studies, such as West et al [30] and Shea et al [4], offered follow-up beyond 12 months.

Sample sizes were frequently small or underpowered (eg, Russell et al [27] and Smith et al [29]), increasing susceptibility to type II errors and reducing the robustness of subgroup analyses. The heterogeneity in intervention types and outcome measures also complicates cross-study comparisons. For example, while weight loss and HbA_1c_ were commonly measured, behavioral metrics varied widely and were often self-reported without standardized instruments.

Additionally, economic evaluations were notably absent. None of the included studies systematically reported on cost-effectiveness, despite the critical importance of economic data for policymaking and reimbursement frameworks in telehealth implementation [33,34]. All included studies were conducted in high-income countries; the absence of eligible studies from low- and middle-income countries limits generalizability. We explicitly searched without country restrictions; the lack of low- and middle-income country evidence appears to reflect publication scarcity rather than an a priori exclusion.

### Limitations of This Review

This review also has several methodological limitations that should be acknowledged. First, the small and heterogeneous evidence base precluded quantitative pooling and limited the generalizability of conclusions. Second, despite a comprehensive search strategy, we restricted inclusion to peer-reviewed, indexed papers, which may have introduced publication and language bias by excluding gray literature and non-English studies. Third, although dual reviewers independently screened and extracted data, subjective judgments in the narrative synthesis may have influenced the interpretation of findings. Finally, all included studies originated from high-income countries, constraining the applicability of results to low- and middle-income settings where digital and infrastructural barriers may differ substantially.

### Barriers to Implementation and Digital Equity

An important contextual finding across studies is the presence of digital and literacy-related barriers among older rural adults [35,36]. Digital literacy was rarely assessed systematically; however, studies like Wilson et al [20] and Smith and Weinert [29] hinted at limited technology familiarity and engagement. Moreover, intervention adherence and satisfaction appeared to be influenced by factors such as perceived complexity, trust in remote systems, and personal contact with health care providers.

These findings echo previous reports emphasizing the need for user-centered, accessible design tailored to the cognitive and sensory needs of older adults. As Barnason et al [16] and DiNapoli et al [18] noted, patient satisfaction and engagement were higher when interventions included regular human interaction, simplified instructions, and consistent technical support.

### Implementation Barriers and Health Equity Considerations

The review highlights persistent digital and infrastructural barriers disproportionately affecting older adults in rural or underserved settings [37,38]. Limited broadband access, low digital literacy, and lack of culturally tailored content not only hinder adoption but reinforce existing health inequities [39-41]. These implementation challenges are structurally linked to social determinants of health, such as lower educational attainment and income levels in rural areas [42]. Without addressing these foundational disparities, digital health solutions risk exacerbating rather than alleviating access gaps. Therefore, policy interventions should include investments in digital infrastructure, community-based training, and participatory design processes that reflect local needs [43].

### Innovation and Emerging Models

This review highlights not only the effectiveness of established telehealth modalities but also the emergence of hybrid care models and novel delivery platforms tailored to rural aging populations. Notably, interventions incorporating both synchronous (eg, phone-based coaching) and asynchronous (eg, mobile tracking or wearable monitoring) modalities were associated with higher patient engagement and adherence. For instance, the Rural Lifestyle, Exercise, Attitudes, and Relationships Program (LEAP) trial [19] compared group versus individual telecoaching, revealing that group-based formats may enhance social connectedness—a crucial factor for isolated older adults. Similarly, the use of wearable-enabled remote monitoring in heart failure management (eg, TIM-HF2 [12], Hózhó trial [7]) represents a shift toward continuous and passive data collection, enabling earlier detection of clinical deterioration.

These findings align with a recent framework such as the National Academies of Sciences, Engineering, and Medicine 2022 Digital Health Equity Roadmap, which emphasizes human-centered design, infrastructure co-development, and inclusive implementation pathways to ensure equitable access to digital health. Compared to earlier reviews that emphasized general digital adoption [31], this study foregrounds context-specific innovations, such as culturally adapted content, caregiver-mediated interfaces, and localized broadband workarounds, which offer blueprints for scalable, equitable intervention design in marginalized aging populations.

### Implications (Practice or Policy)

To translate the observed benefits of telehealth into routine care, integration into existing primary care models is essential [44]. This requires not only technological interoperability but also alignment with reimbursement policies, provider training, and patient education [45]. Interventions led by nurses or care coordinators demonstrated high satisfaction and adherence, suggesting that a team-based, hybrid care model may offer the most sustainable pathway [46]. Health systems must also establish clear protocols for digital triage, follow-up, and escalation of care to ensure clinical safety [47]. Policymakers should incentivize digital inclusion initiatives and consider telehealth reimbursement parity to support widespread adoption in rural and underserved populations [48]. Distinguishing patient-reported outcomes from provider-facing facilitators clarifies that improvements in usability or adherence reflect patient experiences, whereas professional networking and workflow integration represent implementation considerations at the provider/system level.

### Economic Considerations

Although several interventions demonstrated clinical and behavioral benefits, none of the included studies conducted a formal cost-effectiveness analysis. This absence of economic evidence substantially limits the ability of health systems and policymakers to make informed, data-driven decisions regarding large-scale implementation. Without robust cost analyses, it is unclear whether the observed benefits can be achieved sustainably or equitably across diverse settings. Future trials should therefore embed cost-effectiveness and budget impact evaluations alongside clinical outcomes to determine value for money, support reimbursement frameworks, and guide resource allocation in rural and underserved populations.

### Risk of Publication and Language Bias

The small number of included studies limited the feasibility of conducting formal statistical assessments of publication bias, such as funnel plots or regression-based tests. With only 14 trials, these methods would be underpowered and potentially misleading. Nevertheless, the predominance of positive findings raises the possibility of small-study effects. In addition, by restricting our search to peer-reviewed and indexed literature, we may have excluded gray literature or non-English studies that could provide null or negative results. These factors suggest that the overall effectiveness of digital health interventions in rural older populations may be somewhat overestimated, and future reviews should seek to mitigate this risk by incorporating broader data sources and multilingual evidence.

### Recommendations for Future Research

To advance the field, future studies should prioritize longer follow-up durations to assess the sustainability of clinical and behavioral gains. Large, multicenter trials with stratified randomization would enhance generalizability and allow for meaningful subgroup analysis by age, comorbidity burden, or digital literacy level.

Moreover, standardized outcome reporting is critical. Adopting common core sets of metrics—particularly for behavioral and quality-of-life domains—would enable pooled synthesis and meta-analyses, strengthening the evidence base. Economic evaluations, including cost-effectiveness and budget impact analyses, should also be embedded into study designs.

The implementation of satellite-based internet in geographically remote areas with limited or no connectivity can improve the functionality and accessibility of telehealth platforms and applications, facilitating communication between health care providers and older adult patients. In this context, the limited availability of specialist care in underserved regions may be addressed through the progressive integration of telehealth technologies, expanding access to specialized services for aging populations.

Finally, cultural tailoring and language accessibility should be emphasized in designing telehealth platforms. Rural populations are not homogenous; successful interventions must adapt to the social, linguistic, and infrastructural realities of diverse communities.

### Conclusions

Telehealth interventions represent a promising approach to enhancing the health and well-being of older adults in rural and underserved settings. Evidence from 14 studies indicates that remote strategies can contribute to improved management of chronic conditions, support behavioral change, and in some cases, reduce hospitalizations. Yet, important gaps remain regarding cost-effectiveness, outcome standardization, and digital inclusion. Integrating human support with technological solutions appears to enhance adherence and overall effectiveness. Equitable access and long-term sustainability will require greater emphasis on digital literacy, cultural adaptation, and economic feasibility. At present, the lack of formal cost-effectiveness analyses constrains data-driven decisions about large-scale adoption. Future studies should therefore embed economic evaluations and equity considerations to provide the evidence base needed for informed policy and resource allocation in underserved contexts.

## Supplementary material

10.2196/78913 Multimedia Appendix 1 Methodological materials and extended evidence synthesis.

10.2196/78913 Checklist 1 PRISMA checklist.

## Abbreviations

CBT: cognitive behavioral therapy
IDEATel: Informatics for Diabetes Education and Telemedicine
LEAP: Lifestyle, Exercise, Attitudes, and Relationships Program
mHealth: mobile health
NIH: National Institutes of Health
PRISMA: Preferred Reporting Items for Systematic Reviews and Meta-Analyses
PROGRESS-Plus: place of residence; race/ethnicity, occupation, gender, religion, education, socioeconomic status, social capital, plus other context-specific determinants of health equity
PROPS: Population, Remote Health Interventions, Outcomes, Procedures, and Study Design
RoB: Risk of Bias
ROBINS-I: Risk Of Bias In Non-randomized Studies–of Interventions
