# Supplementary material for: Digital and Remote Health for Older Adults in Rural or Underserved Settings: Systematic Review of Clinical, Behavioral, and Implementation Outcomes
Source: JMIR Aging. 2026 Apr 21;9:e78913. doi: 10.2196/78913 (PMC13099032; doi:10.2196/78913)
Supplement: Multimedia Appendix 1 [file aging-v9-e78913-s001.docx]

Multimedia Appendix 1

Supplementary Tables

**Table S1**

| **Database** | **Formulation** | **Filters** |
| --- | --- | --- |
| Pubmed | (  "Rural Population"[MeSH Terms] OR  "Medically Underserved Area"[MeSH Terms] OR  "Health Services Accessibility"[MeSH Terms] OR  "Rural Health"[MeSH Terms] OR  "rural areas"[Title/Abstract] OR  "remote areas"[Title/Abstract] OR  "underserved populations"[Title/Abstract] OR  "geographically isolated"[Title/Abstract] OR  "hard-to-reach areas"[Title/Abstract] OR  "rural communities"[Title/Abstract]  )  AND  (  "Telemedicine"[MeSH Terms] OR  "Teledentistry"[MeSH Terms] OR  "Remote Consultation"[MeSH Terms] OR  "Mobile Health Units"[MeSH Terms] OR  "Electronic Health Records"[MeSH Terms] OR  "Telecommunications"[MeSH Terms] OR  "telehealth"[Title/Abstract] OR  "telemedicine"[Title/Abstract] OR  "teledentistry"[Title/Abstract] OR  "e-health"[Title/Abstract] OR  "mobile health"[Title/Abstract] OR  "teleconsultation"[Title/Abstract] OR  "remote monitoring"[Title/Abstract] OR  "digital health"[Title/Abstract]  )  AND  (  "Treatment Outcome"[MeSH Terms] OR  "Program Evaluation"[MeSH Terms] OR  "Outcome Assessment, Health Care"[MeSH Terms] OR  "Quality of Health Care"[MeSH Terms] OR  "Effectiveness"[Title/Abstract] OR  "treatment outcome"[Title/Abstract] OR  "intervention effectiveness"[Title/Abstract] OR  "clinical effectiveness"[Title/Abstract] OR  "access improvement"[Title/Abstract] OR  "coverage improvement"[Title/Abstract] OR  "service performance"[Title/Abstract] OR  "quality of care"[Title/Abstract] OR  "health service utilization"[Title/Abstract]  ) | Filters applied: Clinical Study, Clinical Trial, Controlled Clinical Trial, Randomized Controlled Trial. |
| Scopus | (TITLE-ABS-KEY("rural area*" OR "remote area*" OR "rural population*" OR "rural communit*" OR "hard-to-reach area*" OR "underserved population*" OR "geographically isolated"))  AND  (TITLE-ABS-KEY("telehealth" OR "telemedicine" OR "teledentistry" OR "e-health" OR "mobile health" OR "teleconsultation" OR "remote monitoring" OR "digital health"))  AND  (TITLE-ABS-KEY("effectiveness" OR "treatment outcome*" OR "intervention effectiveness" OR "clinical effectiveness" OR "program evaluation" OR "healthcare access" OR "access improvement" OR "coverage improvement" OR "health service utilization" OR "service performance" OR "quality of care")) | AND ( LIMIT-TO ( DOCTYPE , "ar" ) ) |
| WoS | TS=("rural area*" OR "remote area*" OR "rural population*" OR "rural communit*" OR "hard-to-reach area*" OR "underserved population*" OR "geographically isolated")  AND  TS=("telehealth" OR "telemedicine" OR "teledentistry" OR "e-health" OR "mobile health" OR "teleconsultation" OR "remote monitoring" OR "digital health")  AND  TS=("effectiveness" OR "treatment outcome*" OR "intervention effectiveness" OR "clinical effectiveness" OR "program evaluation" OR "healthcare access" OR "access improvement" OR "coverage improvement" OR "health service utilization" OR "service performance" OR "quality of care") | Refined By:Document Types: Article |
| Embase | ('rural area*' OR 'remote area*' OR 'rural population*' OR 'rural communit*' OR 'hard-to-reach area*' OR 'underserved population*' OR 'geographically isolated')  AND  ('telehealth' OR 'telemedicine' OR 'teledentistry' OR 'e-health' OR 'mobile health' OR 'teleconsultation' OR 'remote monitoring' OR 'digital health')  AND  ('effectiveness' OR 'treatment outcome*' OR 'intervention effectiveness' OR 'clinical effectiveness' OR 'program evaluation' OR 'healthcare access' OR 'access improvement' OR 'coverage improvement' OR 'health service utilization' OR 'service performance' OR 'quality of care') | AND 'randomized controlled trial'/de |
| Cochrane Library | ("rural area*" OR "remote area*" OR "rural population*" OR "hard-to-reach area*" OR "underserved population*" OR "geographically isolated")  AND  ("telehealth" OR "telemedicine" OR "teledentistry" OR "e-health" OR "mobile health" OR "teleconsultation" OR "remote monitoring" OR "digital health")  AND  ("effectiveness" OR "treatment outcome*" OR "program evaluation" OR "access improvement" OR "coverage improvement" OR "health service utilization" OR "quality of care") |  |

**Table S2. Evidence profile by outcome domain**

| **Study** | **Domain** | **Primary outcome** | **Effect direction** | **Magnitude (Δ or RR)** | **Precision (95% CI / P)** | **Certainty (narrative)** |
| --- | --- | --- | --- | --- | --- | --- |
| Barnason et al. (2019) | Clinical (weight) | Weight loss | ↓ weight vs control | Mean Δ –2.5 kg at 6 mo | P<0.05 | Moderate |
|  | Behavioral | Self-efficacy | ↑ activity, adherence | Not reported | P<0.05 | Moderate |
| DiNapoli et al. (2017) | Behavioral | Anxiety/depression | ↓ symptoms | Δ –3.2 PHQ-9 | P<0.05 | Moderate |
|  | Psychosocial | QoL | ↑ QoL scores | Not reported | Not reported | Moderate |
| Eberly et al. (2025) | Clinical | GDMT uptake | ↑ GDMT initiation | +18% vs control | P<0.01 | High |
|  | Utilization | HF hospitalization | ↓ 30-day readmissions | OR 0.62 | 95% CI (0.41–0.95) | High |
| Krum et al. (2013) | Utilization | Hospitalizations | ↓ all-cause admissions | HR 0.76 | P=0.04 | Moderate |
| Lear et al. (2015) | Clinical | Lipid profile | ↓ LDL | Δ –12 mg/dL | P<0.05 | Moderate |
| Perri et al. (2019) | Clinical | Weight loss | Sustained ↓ | Mean Δ –5.8 kg at 12 mo | P<0.01 | High |
|  | Behavioral | Goal attainment | ↑ adherence | Not reported | P<0.01 | High |
| Perri et al. (2020) | Clinical | Weight loss | Sustained ↓ | Δ –4.9 kg at 16 mo | P<0.01 | High |
|  | Behavioral | Maintenance | ↑ long-term adherence | Not reported | Not reported | Moderate |
| Prescher et al. (2023) | Clinical | Cardiac function | ↑ function, QoL | Δ 0.35 (QoL index) | P<0.05 | High |
|  | Utilization | Hospitalizations | ↓ admissions | Not reported | Not reported | Moderate |
| Russell et al. (2011) | Clinical | OA outcomes | ↑ function | Δ WOMAC –8 pts | P<0.05 | Moderate |
|  | Utilization | Acute episodes | ↓ episodes | Not reported | Not reported | Low |
| Shea et al. (2009) | Clinical | HbA1c, BP, LDL | ↓ HbA1c (–0.8%), ↓ SBP (–6 mmHg), ↓ LDL (–14 mg/dL) | 95% CI not reported | P<0.01 | High |
| Shea et al. (2013) | Clinical | HbA1c, SBP | ↓ HbA1c (–0.5%), ↓ SBP (–5 mmHg) | P<0.05 | Moderate |  |
| Smith et al. (2000) | Behavioral | Health awareness | ↑ awareness | Not quantified | Not reported | Low |
| West et al. (2010) | Clinical | Diabetes control | Improved glycemic indices | Not quantified | Not reported | Moderate |
|  | Behavioral | Goal execution | ↑ goal attainment | Not reported | Not reported | Low |
| Wilson et al. (2016) | Clinical | Smoking cessation | ↑ abstinence rates | 20% vs 8% control | P<0.05 | Moderate |
|  | Behavioral | Self-efficacy | ↑ abstinence confidence | Not reported | Not reported | Moderate |

**Table S3. Risk-of-bias domain frequencies across included studies (N=14)**

| **Tool / Domain** | **Low risk n/N (%)** | **Some concerns n/N (%)** | **High risk n/N (%)** |
| --- | --- | --- | --- |
| **RoB 2 (n=7 RCTs)** |  |  |  |
| Randomization process | 5/7 (71%) | 2/7 (29%) | 0/7 (0%) |
| Deviations from intended interventions | 4/7 (57%) | 3/7 (43%) | 0/7 (0%) |
| Missing outcome data | 3/7 (43%) | 3/7 (43%) | 1/7 (14%) |
| Measurement of outcomes | 5/7 (71%) | 2/7 (29%) | 0/7 (0%) |
| Selection of reported result | 3/7 (43%) | 3/7 (43%) | 1/7 (14%) |
| **ROBINS-I (n=4 non-randomized)** |  |  |  |
| Confounding | 1/4 (25%) | 2/4 (50%) | 1/4 (25%) |
| Selection of participants | 2/4 (50%) | 2/4 (50%) | 0/4 (0%) |
| Classification of interventions | 2/4 (50%) | 2/4 (50%) | 0/4 (0%) |
| Deviations from intended interventions | 2/4 (50%) | 1/4 (25%) | 1/4 (25%) |
| Missing data | 1/4 (25%) | 2/4 (50%) | 1/4 (25%) |
| Measurement of outcomes | 2/4 (50%) | 2/4 (50%) | 0/4 (0%) |
| Selection of reported result | 1/4 (25%) | 2/4 (50%) | 1/4 (25%) |
| **NIH Quality Assessment (n=3 observational)** |  |  |  |
| Study participation | 2/3 (67%) | 1/3 (33%) | 0/3 (0%) |
| Outcome measurement | 2/3 (67%) | 1/3 (33%) | 0/3 (0%) |
| Confounding control | 1/3 (33%) | 1/3 (33%) | 1/3 (33%) |
| Attrition | 2/3 (67%) | 0/3 (0%) | 1/3 (33%) |

**Table S4. PROGRESS-Plus Equity Appraisal of Included Studies (N=14)**

| **Study** | **Place** | **Race/Ethnicity** | **Occupation** | **Gender** | **Religion** | **Education** | **SES** | **Social capital** | **Plus** |
| --- | --- | --- | --- | --- | --- | --- | --- | --- | --- |
| Barnason 2019 | Rural (USA) | NR | NR | Reported | NR | NR | NR | NR | Overweight/obese post-PCI or CABG |
| DiNapoli 2017 | Rural (USA) | Ethnically diverse | NR | Reported | NR | Reported | Low-resource | NR | Mild cognitive impairment, frailty |
| Eberly 2025 | Underserved reservations (USA) | 100% American Indian | NR | Reported | NR | NR | Medicare/Medicaid | NR | Age 62–74; diabetes, AFib, CKD |
| Krum 2013 | Rural/remote/outer metro (AUS) | NR | NR | Reported | NR | NR | NR | NR | HF patients, mean age 73, comorbidities |
| Lear 2015 | Rural & small urban (Canada) | NR | NR | Reported | NR | NR | NR | NR | Post-cardiac rehab |
| Perri 2019 | Rural Florida (USA) | Majority White, some minorities | NR | Reported | NR | Reported | Low-income | NR | Obesity BMI 30–45 |
| Perri 2020 | 14 rural counties Florida (USA) | 74% White, 19% Black, 4% Hispanic | NR | 82.7% women | NR | 54% ≥12 yrs education | 50% <50k income | NR | Obesity, mean age 55 |
| Prescher 2023 | Rural vs urban (Germany) | NR | NR | 70% men | NR | NR | NR | NR | Age 71.5, HF NYHA II–III |
| Russell 2011 | Rural/remote (Australia) | NR | NR | 41–61% women | NR | NR | NR | NR | Post-TKA, mean age 68 |
| Shea 2009 | Underserved NY (USA) | 15% Black, 36% Hispanic, 48% White | NR | 63% women | NR | Mean 9.7 yrs | 39% Medicaid | NR | Older adults, low digital literacy |
| Shea 2013 | Underserved NY (USA) | 15% Black, 36% Hispanic, 48% White | NR | 63% women | NR | 0–13+ yrs (varied) | 39% Medicaid, mean $15k | NR | Explicit SES analysis, low literacy |
| Smith 2000 | Rural USA | NR | NR | Reported | NR | NR | NR | NR | Older adults, low QoL |
| West 2010 | Rural USA | NR | NR | Reported | NR | NR | NR | NR | Older adults with diabetes, 2–6 yr follow-up |
| Wilson 2016 | Rural veterans USA | 56% Black, 38% White, 5% Multiracial, 1% AI/AN, 1% Hispanic | 52% disabled, 26% retired, 23% employed | 11% women | NR | 7% <HS, 27% HS, 39% some college, 27% college+ | Low SES (many disabled/unemployed) |  |  |

**Table S5. Adherence and Attrition Across Included Studies (N=14)**

| **Study** | **Adherence definition** | **Adherence (%)** | **Drop-out (%)** | **Reasons for attrition** |
| --- | --- | --- | --- | --- |
| Barnason 2019 | Completion of scheduled phone coaching sessions | ~75% | 18% | Scheduling conflicts, loss of interest |
| DiNapoli 2017 | Attendance at ≥80% of CBT sessions | 82% | 15% | Cognitive decline, transportation barriers |
| Eberly 2025 | Uptake and continuation of GDMT titration and monitoring | 88% | 10% | Patient relocation, technical barriers |
| Krum 2013 | Participation in remote monitoring calls | 70% | 25% | Equipment difficulties, comorbid illness |
| Lear 2015 | Log-ins and completion of online rehab modules | 72% | 20% | Limited internet access, technical difficulties |
| Perri 2019 | Completion of telecoaching sessions and self-monitoring logs | 84% | 16% | Work/family responsibilities, loss of motivation |
| Perri 2020 | Participation in group/individual phone counseling, session attendance | 87% | 13% | Competing demands, internet/phone barriers |
| Prescher 2023 | Continuous use of remote monitoring devices over 12 mo | 90% | 12% | Device fatigue, technical breakdown |
| Russell 2011 | Participation in telerehab exercises and reporting | 80% | 15% | Post-surgical complications, connectivity |
| Shea 2009 | Completion of home telemonitoring data uploads | 78% | 22% | Lack of computer literacy, equipment malfunction |
| Shea 2013 | Consistent data transmission and case management calls | 76% | 24% | Low digital literacy, attrition from cohort |
| Smith 2000 | Attendance to telehealth counseling sessions | 70% | 20% | Lack of interest, mobility problems |
| West 2010 | Participation in telehealth diabetes management over 2–6 yrs | ~60% at 2 yrs | 40% | Long-term fatigue, loss to follow-up |
| Wilson 2016 | Completion of scheduled telehealth smoking cessation sessions | 74% | 21% | Psychiatric comorbidities, relapse, technical issues |

**Table S6 – Intervention Models and Applicability**

| **Intervention model** | **Delivery type** | **Clinical domain(s)** | **Example studies** | **Observed effects** | **Applicability** |
| --- | --- | --- | --- | --- | --- |
| **Synchronous, single-component** | Real-time phone or video sessions | Mental health, smoking cessation | DiNapoli 2017; Wilson 2016 | Improved mood, cessation rates, patient engagement | Useful for targeted behavioral support in small populations |
| **Synchronous, multicomponent** | Phone monitoring + nurse case management | Diabetes, heart failure | Shea 2009, 2013; Eberly 2025 | HbA1c ↓, BP ↓, higher GDMT uptake, reduced HF hospitalizations | Effective for chronic disease management; requires staff training |
| **Asynchronous, single-component** | Web-based modules or telerehab programs | Cardiac rehab, post-surgical recovery | Lear 2015; Russell 2011 | Improved exercise capacity, functional outcomes | Feasible where broadband access is reliable |
| **Asynchronous, multicomponent** | Online tracking + coaching, remote monitoring + care coordination | Obesity, cardiac conditions | Perri 2019, 2020; Prescher 2023 | Sustained weight loss, improved motivation, reduced admissions | Supports long-term adherence; scalable with digital literacy support |
